# TRPV1, a novel biomarker associated with lung cancer via excluding immune infiltration

**DOI:** 10.1002/mco2.139

**Published:** 2022-05-23

**Authors:** Rui Gao, Mei Meng, Xianchao Zhou, Miao Yu, Zhifan Li, Jingquan Li, Xiaonan Wang, Yizhi Song, Hui Wang, Jian He

**Affiliations:** ^1^ State Key Laboratory of Oncogenes and Related Genes Center for Single‐Cell Omics School of Public Health Shanghai Jiao Tong University School of Medicine Shanghai China; ^2^ Division of Life Sciences and Medicine, School of Biomedical Engineering (Suzhou) University of Science and Technology of China Suzhou China; ^3^ CAS Key Laboratory of Bio‐Medical Diagnostics Suzhou Institute of Biomedical Engineering and Technology Chinese Academy of Sciences Suzhou China; ^4^ Shanghai Jiao Tong University School of Medicine Shanghai China

1

Dear Editor,

Lung cancer is one of the most common malignant tumors worldwide, and its metastasis is a key biological process causing poor prognosis. In the past two decades, lung adenocarcinoma (LUAD) has been referred to as a type of nonimmunogenic cancer.^1^ However, along with the increasing understanding of tumor‐immune interactions, this model in lung cancer was questioned.^2^ The mechanisms of immune‐related interaction play key roles in the genesis and development of tumors, and immunotherapy is considered to be a promising approach for cancer therapeutics.^3^ Note that 2021 Medicine Nobel went to scientists who discovered the biology of  TRPV1, which helps researchers to understand the molecular basis for pain.^4^ But few research has elucidated the role of TRPV1 in malignancies, especially the correlations of TRPV1 to the prognoses and tumor‐infiltrating lymphocytes (TILs) in different cancers remain poorly described.^5^ Here, we aimed to assess TRPV1 as well as a potential prognostic biomarker and to explore its possibility to become a novel therapeutic target in lung cancer.

Firstly, we analyzed the levels of TRPV1 mRNA expression using the online RNA‐Seq datasets of multiple tumor types. Compared to the paired adjacent normal tissues, the mRNA expression level of TRPV1 in the tumor tissues of LUAD and LUSC was significantly higher (Figure 1A). Secondly, we investigated whether TRPV1 mRNA expression level was associated with the prognosis. It is shown that the higher the expression level of TRPV1, the worse the prognosis (Figure 1B). Therefore, we believe that the high expression level of TRPV1 mRNA is an independent risk factor for poor prognosis in lung cancer patient cohorts, and HR higher than 1 indicates that the mRNA expression of this gene could be a risk factor. Meanwhile, the different prognosis patterns between LUAD and LUSC caught our attention (Figure 1C,D). These statistical results indicated the prognostic potential of TRPV1 in lung cancer, and the difference in TRPV1 expression leads to different prognostic potential patterns in different subtypes of lung cancer.

**FIGURE 1:**
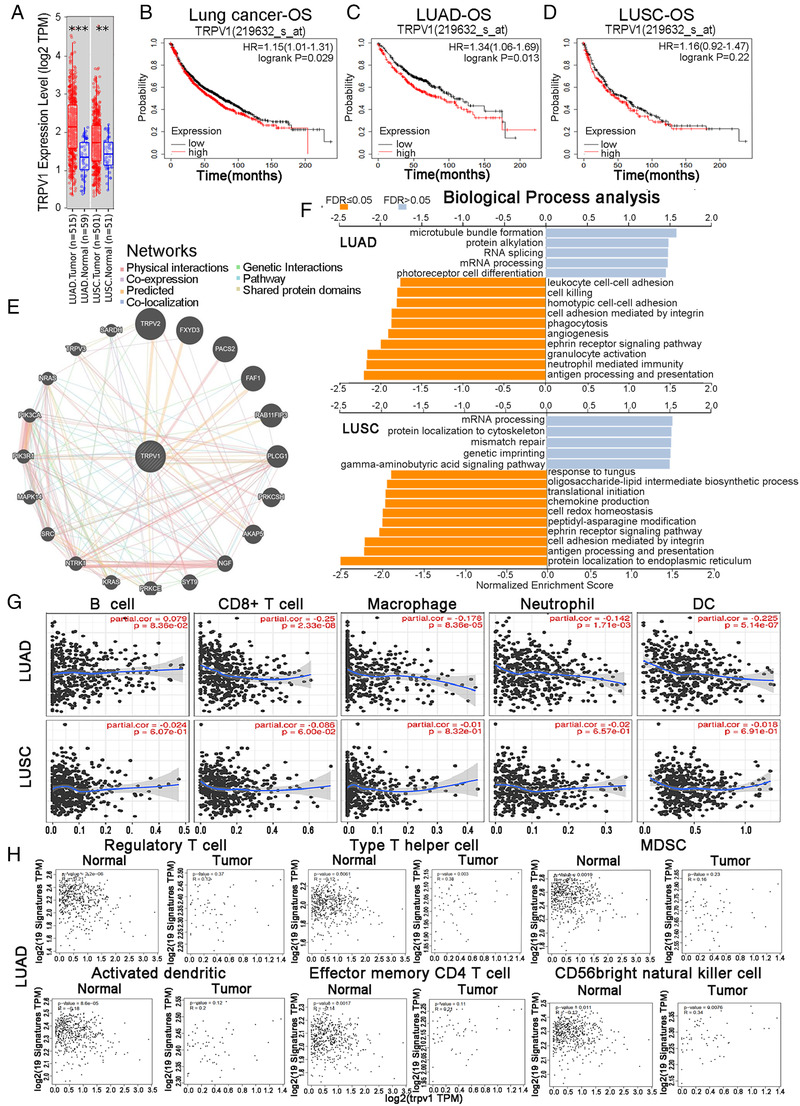
(A) The varied expression levels between tumor and adjacent normal tissues for TRPV1 across two different types of lung cancer (lung adenocarcinoma [LUAD] and LUSC). Survival curves of OS in (B) lung cancer, (C) LUAD and (D) LUSC. (E) Biological interaction network of TRPV1. TRPV1 interaction network in TCGA, different colors represent diverse bioinformatics methods. (F) Enriched Gene Ontology annotations of biological process analysis of TRPV1 correlated genes in LUAD (upper) and LUSC (lower). Dark blue and orange indicate FDR ≤ 0.05, light blue, and orange indicate FDR > 0.05. FDR, false discovery rate. (G) Correlation of TRPV1 expression with immune infiltration level in LUAD and LUSC. TRPV1 expression is significantly negatively correlated the level of CD8+ T cells, macrophages, neutrophils, and DCs in LUAD (*n* = 515) rather than that in LUSC (*n* = 501). (H) Correlation analysis between TRPV1 expression and various immune cells in normal and tumor tissue of LUAD. Scatterplots of correlations between TRPV1 expression and regulatory T cell, Type 2 T helper cell, MDSC, activated dendritic cell, activated B cell, eosinophil, effector memory CD4 T cell, CD56 bright natural killer cell in the normal and tissue of LUAD (HR, hazard ratio, PFS, progression‐free survival, TME, tumor microenviroment, LUSC, lung squamous cell cancer, LUAD, lung adenocarcinoma, OS, overall survival, TCGA, The Cancer Genome Atlas, FDR, false discovery rate, DCs, dendritic cell, MDSC, myeloid‐derived suppressor cells)

Also, we determined the correlation between TRPV1 mRNA expression levels and various clinical features, in order to better elucidate the correlation and mechanism of the function of TRPV1 in lung cancer, especially the lung cancer patients in different clinical stages. It is shown that higher mRNA expression of TRPV1 was correlated to poorer OS and PFS in female lung cancer patients while only associated with poor PFS in male patients (Table S1). High levels of TRPV1 mRNA were associated with poor OS in the early stages (stages 1 and 2) rather than that in the late stage (Table S1). Furthermore, we analyzed these factors between LUAD and LUSC cohorts. It is found that TRPV1 mRNA expression level was associated with the poor prognostic potential of LUAD patient cohorts in most of these factors we discussed above (Tables S2 and S3). This striking phenomenon is that the divergent OS patterns of LUAD and LUSC may refer to the correlation between TRPV1 and the prognosis of various types of cancer, depending on the diverse mechanisms of tumor initiation and progression.

An interaction network showed that TRPV1 has co‐expression with 20 proteins, and most of these proteins are cancer‐associated proteins (Figure 1E). Volcano plot showing top 50 correlated positive and negative genes (Figure S1). These data indicate that TRPV1 takes an important part in the development of lung cancer. Gene set enrichment analysis was used to analyze biological process, and then we found that TRPV1 related to differential expressed genes was connected with a variety of immunobiological processes, including “antigen processing and presentation,” “neutrophil‐mediated pathway,” “granulocyte activation,” “interleukin‐12‐related response,” “response to interferon‐gamma,” “interleukin‐6 production,” “interferon‐gamma production,” “leukocyte migration,” and “cytokine secretion” in LUAD patient cohorts rather than in LUSC patient cohorts (Figure 1F). All these results above suggested that TRPV1 plays a crucial role in immune‐activating functions in lung cancer, especially in LUAD.

TILs have been demonstrated to be independent predictor of various cancers.^4^ It is shown that TRPV1 mRNA expression levels were associated with poor OS and excluding immune‐infiltrating in LUAD patients but not in LUSC patients. The expression level of TRPV1 was significantly negatively correlated with the infiltration levels of CD8+ T cell, macrophages, neutrophils, and DCs in LUAD (Figure 1G). These findings powerfully indicate that TRPV1 takes a specific part in immune infiltration in LUAD and LUSC and causes a poor prognosis in LUAD patients rather than in LUSC patients.

To analyze the correlation between TRPV1 and various types of tumor‐infiltrating immune cells, we concentrated on the association between the expression level of TRPV1 mRNA and immune marker sets of each type of immunocytes. We found that the expression level of TRPV1 mRNA level was significantly negatively correlated with some immune marker sets and nonsignificant correlation of different types of immune cells, including monocytes and different T cell subtypes, especially the effect T‐cells in LUAD patient cohorts (Table S4). However, among these gene marker sets no one was significantly negatively connected with the TRPV1 mRNA expression level in LUSC patients and even more, some other immune marker genes are positively correlated (Table S4). These data showed that the marker genes’ mRNA expression levels in T‐cells (CD8+, effector T, resident memory T, naive T), monocyte, tumor‐associated macrophage, M1 macrophages, natural killer cell, and DCs were negatively correlated to the TRPV1 mRNA expression level in LUAD cohorts (Table S4, Figure S2). Furthermore, we found that NOS2, PTGS2, and IRF5 of M1 phenotype are nonsignificantly correlated with TRPV1 mRNA level in LUAD, while IRF5 is significantly positive with TRPV1 expression (Table S4). M1 has been reported to prevent tumor development. Whether TRPV1 is an important factor in macrophage depolarization mediation, and tumor microenvironment remodeler requires in‐depth study. (Table S4). Also, we compared the expression level of TRPV1 with marker sets of monocytes and various types of T‐cells in cancer tissue and normal tumor‐adjacent tissue of patients with LUAD or LUSC and further revealed their associations. We were surprised to find that the mRNA levels of top genes of the marker sets of these immunocytes were strongly negatively correlated with TRPV1 in the cancer tissue of LUAD patients (Figure 1H). In the LUSC patient cohorts, TRPV1 had no negative significant correlation with immunocytes except MDSC; moreover, the correlation of the TRPV1 expression level and the gene markers of CD56 bright natural killer cell in LUSC patient cohorts was significantly positive (Figure S3). These findings might imply that underlying correlation patterns between LUAD and LUSC, as well as the cancer tissue and normal tumor‐adjacent tissue of LUAD patients. These exhilarating findings suggested that TRPV1 might exclude various types of immune cells in the TME of the LUAD, and TRPV1 might be a novel and potential therapeutic target for LUAD therapy. These results further indicate that there might be a robust relationship between TRPV1 mRNA level and monocytes/DCs dual‐exclusion.

In summary, we elucidated that TRPV1 expression was upregulated in cancer tissue of LUAD patients and significantly negatively correlated to the OS of LUAD patients. All these further confirm that TRPV1 is specifically associated with excluding TILs, which implies that TRPV1 plays a crucial part in excluding immunocytes in LUAD. Our findings suggest that TRPV1 might be a new promising target for LUAD therapy due to its modulation of the TME.

## CONFLICT OF INTEREST

All the authors declare there is no conflict of interest.

## AUTHOR CONTRIBUTIONS

*Methodology, reviewing, and editing*: Rui Gao, Mei Meng, Xiaochao Zhou, and Miao Yu. *Data curation*: Zhifan Li, Jingquan Li, and Xiaonan Wang. *Visualization*: Yizhi Song. *Supervision*: Hui Wang. *Conceptualization and writing ‐ original draft*: Jian He.

## ETHICS STATEMENT

This project was permitted and approved by the Ethics Committee of Shanghai Jiao Tong University School of Medicine.

## Supporting information

Supporting information

## Data Availability

All data are available from the corresponding author upon request.
