# Supplementary material for: TRPV1, a novel biomarker associated with lung cancer via excluding immune infiltration
Source: MedComm (2020). 2022 May 23;3(2):e139. doi: 10.1002/mco2.139 (PMC9126026; doi:10.1002/mco2.139)
Supplement: Supplementary file 1 — Supporting information [file MCO2-3-e139-s001.docx]

**Supplementary material**

**TRPV1, a potential biomarker associated with lung cancer via excluding immune infiltration**

Rui Gao^1#^, Mei Meng^1#^, Xianchao Zhou^1#^, Miao Yu^2, 3#^, Zhifan Li^4^, Jingquan Li^1^, Xiaonan Wang^1^, Yizhi Song^2, 3⁎^, Hui Wang^1⁎^, Jian He^1⁎^

*^1^ State Key Laboratory of Oncogenes and Related Genes, Center for Single-Cell Omics, School of Public Health, Shanghai Jiao Tong University School of Medicine, Shanghai 200025, China.*

*^2^ CAS Key Laboratory of Bio-Medical Diagnostics, Suzhou Institute of Biomedical Engineering and Technology, Chinese Academy of Sciences, Suzhou 215163*

*^3^ Division of Life Sciences and Medicine, School of Biomedical Engineering (Suzhou), University of Science and Technology of China, Suzhou 215163, China.*

*^4^ Shanghai Jiao Tong University School of Medicine, Shanghai 200025, China.*

^#^ Equal contribution

^⁎^ Correspondence to: Yizhi Song, Hui Wang and Jian He

**Running Title: Novel lung cancer prognostic biomarker TRPV1**

Table S1 | Correlation of the mRNA expression level of TRPV1 in different stage and clinical prognostic potential in lung cancer with different clinicopathological factors. (219632_s_at)

| **Clinicopathological Characteristics** | **Lung Cancer** | | | | | |
| --- | --- | --- | --- | --- | --- | --- |
|  | **Overall survival (n = 726)** | | | Progression Free Survival (n = 982) | | |
|  | N | Hazard ratio | P-value | N | Hazard ratio | P-value |
| **Sex** | | | | | | |
| Female | 714 | 1.34 (1.05–1.7) | **0.017** | 468 | 1.67 (1.25-2.23) | **0.00047** |
| Male | 1100 | 1.1 (0.93-1.31) | 0.26 | 514 | 1.53 (1.18-1.99) | **0.0014** |
| **Smoking history** | | | | | | |
| Never | 205 | 2.99 (1.71-5.23) | **5.3E-05** | 193 | 1.87 (1.16-3.03) | **0.0095** |
| Smoker | 820 | 1.4 (1.13-1.73) | **0.0018** | 603 | 1.46 (1.14-1.88) | **0.0026** |
| **Stage** | | | | | | |
| 1 | 577 | 1.48 (1.11-1.96) | **0.0064** | 325 | 1.11 (0.7-1.77) | 0.65 |
| 2 | 244 | 1.61 (1.1-2.37) | **0.013** | 130 | 0.81 (0.46-1.43) | 0.48 |
| 3 | 70 | 0.9 (0.5-1.62) | 0.72 | 19 | --- | --- |
| 4 |  | --- | --- |  | --- | --- |

*Bold values indicate P* < *0.05.*

Table S2 | Correlation of the mRNA expression level of TRPV1 in different stage and clinical prognostic potential in LUAD and LUSC with different clinicopathological factors. (219632_s_at)

| **Clinicopathological Characteristics** | **Overall survival (n = 1925)** | | | | | |
| --- | --- | --- | --- | --- | --- | --- |
|  | **LUAD (n= 719)** | | | **LUSC (n= 524)** | | |
|  | N | Hazard ratio | P-value | N | Hazard ratio | P-value |
| **Sex** | | | | | | |
| Female | 317 | 1.94 (1.33–2.85) | **0.00054** | 129 | 1.31 (0.74-2.3) | 0.35 |
| Male | 344 | 1.21 (0.87-1.71) | 0.26 | 342 | 1.15 (0.85-1.56) | 0.37 |
| **Smoking history** | | | | | | |
| Never | 143 | 0.83 (0.34-2.01) | 0.68 | 9 | --- | --- |
| Smoker | 246 | 1.42 (0.88-2.3) | 0.15 | 244 | 1.41 (0.96-2.06) | 0.075 |
| **Stage** | | | | | | |
| 1 | 370 | 1.76 (1.19 -2.6 ) | **0.004** | 172 | 1.35 (0.85-2.16) | 0.21 |
| 2 | 136 | 1.78 (1.08-2.93) | **0.021** | 100 | 1.77 (0.97-3.24) | 0.059 |
| 3 | 24 | 1.23 (0.38-3.99) | 0.73 | 43 | 0.88 (0.43-1.82) | 0.74 |
| 4 |  | --- | --- |  | --- | --- |
| **Treatment** | | | | | | |
| Surgery success  (Surgical margin neg) | 204 | 0.07 (0.01-0.49) | **4E-04** | 65 | 0.52 (0.21-1.29) | 0.15 |

*Bold values indicate P* < *0.05.*

**Table S3 | T**R**PV1** **impacts the clinicopathological parameters of lung cancer patients (LUAD & LUSC)**

| **LUAD Sample types** |  |
| --- | --- |
| Comparison | Statistical significance |
| Normal-vs-Primary | 2.12E-01 |
|  |  |
| **LUAD Individual cancer stage** |  |
| Comparison | Statistical significance |
| Normal-vs-Stage1 | 2.51E-01 |
| Normal-vs-Stage2 | 2.17E-01 |
| Normal-vs-Stage3 | 6.67E-02 |
| Normal-vs-Stage4 | 8.37E-01 |
| Stage1-vs-Stage2 | 8.59E-01 |
| Stage1-vs-Stage3 | 2.71E-01 |
| Stage1-vs-Stage4 | 4.27E-01 |
| Stage2-vs-Stage3 | 3.59E-01 |
| Stage2-vs-Stage4 | 4.03E-01 |
| Stage3-vs-Stage4 | 2.54E-01 |
|  |  |
| **LUAD Patient’s race** |  |
| Comparison | Statistical significance |
| Normal-vs-Caucasian | 1.70E-01 |
| Normal-vs-AfricanAmerican | 8.88E-01 |
| Normal-vs-Asian | 2.48E-01 |
| Caucasian-vs-AfricanAmerican | 1.34E-01 |
| Caucasian-vs-Asian | 4.54E-01 |
| AfricanAmerican-vs-Asian | 2.20E-01 |
|  |  |
| **LUAD Patient’s gender** |  |
| Comparison | Statistical significance |
| Normal-vs-Male | 5.39E-01 |
| Normal-vs-Female | 7.86E-02 |
| Male-vs-Female | 2.94E-02 |
|  |  |
| **LUAD Patient’s age** |  |
| Comparison | Statistical significance |
| Normal-vs-Age(21-40Yrs) | 7.72E-01 |
| Normal-vs-Age(41-60Yrs) | 1.28E-01 |
| Normal-vs-Age(61-80Yrs) | 1.72E-01 |
| Normal-vs-Age(81-100Yrs) | 5.55E-01 |
| Age(21-40Yrs)-vs-Age(41-60Yrs) | 3.10E-01 |
| Age(21-40Yrs)-vs-Age(61-80Yrs) | 3.64E-01 |
| Age(21-40Yrs)-vs-Age(81-100Yrs) | 9.82E-01 |
| Age(41-60Yrs)-vs-Age(61-80Yrs) | 7.32E-01 |
| Age(41-60Yrs)-vs-Age(81-100Yrs) | 3.11E-02 |
| Age(61-80Yrs)-vs-Age(81-100Yrs) | 4.74E-02 |
|  |  |
| **LUAD Smoking status** |  |
| Comparison | Statistical significance |
| Normal-vs-NonSmoker | 1.88E-01 |
| Normal-vs-Smoker | 1.18E-01 |
| Normal-vs-ReformedSmoker1 | 3.03E-01 |
| Normal-vs-ReformedSmoker2 | 3.97E-01 |
| NonSmoker-vs-Smoker | 9.22E-01 |
| NonSmoker-vs-ReformedSmoker1 | 5.63E-01 |
| NonSmoker-vs-ReformedSmoker2 | 3.00E-01 |
| Smoker-vs-ReformedSmoker1 | 4.18E-01 |
| Smoker-vs-ReformedSmoker2 | 1.82E-01 |
| ReformedSmoker1-vs-ReformedSmoker2 | 6.18E-01 |
|  |  |
| **LUAD Nodal metastasis status** |  |
| Comparison | Statistical significance |
| Normal-vs-N0 | 2.36E-01 |
| Normal-vs-N1 | 2.93E-01 |
| Normal-vs-N2 | 1.71E-01 |
| Normal-vs-N3 | N/A |
| N0-vs-N1 | 9.30E-01 |
| N0-vs-N2 | 7.10E-01 |
| N0-vs-N3 | N/A |
| N1-vs-N2 | 6.81E-01 |
| N1-vs-N3 | N/A |
| N2-vs-N3 | N/A |
|  |  |
| **LUAD TP53 mutation status** |  |
| Comparison | Statistical significance |
| Normal-vs-TP53 Mutant | 1.59E-01 |
| Normal-vs-TP53 NonMutant | 2.81E-01 |
| TP53 Mutant-vs-TP53 NonMutant | 3.00E-01 |

| **LUSC Sample types** |  |
| --- | --- |
| Comparison | Statistical significance |
| Normal-vs-Primary | 9.85E-09 |
|  |  |
| **LUSC Individual cancer stage** | |
| Comparison | Statistical significance |
| Normal-vs-Stage1 | 2.08E-06 |
| Normal-vs-Stage2 | 2.86E-03 |
| Normal-vs-Stage3 | 7.88E-03 |
| Normal-vs-Stage4 | 3.55E-01 |
| Stage1-vs-Stage2 | 1.82E-01 |
| Stage1-vs-Stage3 | 1.62E-01 |
| Stage1-vs-Stage4 | 1.04E-02 |
| Stage2-vs-Stage3 | 9.32E-01 |
| Stage2-vs-Stage4 | 5.44E-02 |
| Stage3-vs-Stage4 | 3.24E-01 |
|  |  |
| **LUSC Patient’s race** |  |
| Comparison | Statistical significance |
| Normal-vs-Caucasian | 1.74E-06 |
| Normal-vs-AfricanAmerican | 1.60E-03 |
| Normal-vs-Asian | 6.71E-01 |
| Caucasian-vs-AfricanAmerican | 2.63E-01 |
| Caucasian-vs-Asian | 2.39E-02 |
| AfricanAmerican-vs-Asian | 4.62E-02 |
|  |  |
| **LUSC Patient’s gender** |  |
| Comparison | Statistical significance |
| Normal-vs-Male | 3.52E-07 |
| Normal-vs-Female | 2.38E-03 |
| Male-vs-Female | 6.90E-01 |
|  |  |
| **LUSC Patient’s age** |  |
| Comparison | Statistical significance |
| Normal-vs-Age(21-40Yrs) | N/A |
| Normal-vs-Age(41-60Yrs) | 3.72E-02 |
| Normal-vs-Age(61-80Yrs) | 9.11E-08 |
| Normal-vs-Age(81-100Yrs) | 1.68E-01 |
| Age(21-40Yrs)-vs-Age(41-60Yrs) | N/A |
| Age(21-40Yrs)-vs-Age(61-80Yrs) | N/A |
| Age(21-40Yrs)-vs-Age(81-100Yrs) | N/A |
| Age(41-60Yrs)-vs-Age(61-80Yrs) | 3.77E-01 |
| Age(41-60Yrs)-vs-Age(81-100Yrs) | 3.17E-01 |
| Age(61-80Yrs)-vs-Age(81-100Yrs) | 4.04E-01 |
|  |  |
| **LUSC Smoking status** |  |
| Comparison | Statistical significance |
| Normal-vs-NonSmoker | 1.80E-01 |
| Normal-vs-Smoker | 2.30E-06 |
| Normal-vs-ReformedSmoker1 | 2.11E-05 |
| Normal-vs-ReformedSmoker2 | 1.91E-02 |
| NonSmoker-vs-Smoker | 5.68E-01 |
| NonSmoker-vs-ReformedSmoker1 | 8.12E-01 |
| NonSmoker-vs-ReformedSmoker2 | 8.05E-01 |
| Smoker-vs-ReformedSmoker1 | 2.28E-01 |
| Smoker-vs-ReformedSmoker2 | 1.99E-01 |
| ReformedSmoker1-vs-ReformedSmoker2 | 4.26E-01 |
|  |  |
| **LUSC Nodal metastasis status** | |
| Comparison | Statistical significance |
| Normal-vs-N0 | 2.69E-07 |
| Normal-vs-N1 | 1.14E-02 |
| Normal-vs-N2 | 5.06E-02 |
| N0-vs-N1 | 6.22E-01 |
| N0-vs-N2 | 5.67E-01 |
| N1-vs-N2 | 8.93E-01 |
|  |  |
| **LUSC TP53 mutation status** | |
| Comparison | Statistical significance |
| Normal-vs-TP53 Mutant | 2.97E-07 |
| Normal-vs-TP53 NonMutant | 6.99E-04 |
| TP53 Mutant-vs-TP53 NonMutant | 4.07E-01 |

**Table S4 | Correlation analysis between TRPV1 and relate markers of immune cells.**

| **Description** | **Gene Markers** | **LUAD** | | | | **LUSC** | | | |
| --- | --- | --- | --- | --- | --- | --- | --- | --- | --- |
|  |  | **None** | | **Purity** | | **None** | | **Purity** | |
|  |  | **Cor** | ***p*** | **Cor** | ***p*** | **Cor** | ***p*** | **Cor** | ***p*** |
| CD8+ T cell | CD8A | -0.084 | 0.057 | -0.077 | 0.087 | -0.013 | 0.766 | 0.055 | 0.230 |
|  | CD8B | -0.077 | 0.081 | -0.072 | 0.112 | 0.009 | 0.849 | 0.052 | 0.252 |
| T cell (general) | CD3D | -0.122 | * | -0.115 | 0.010 | -0.079 | 0.077 | 0.005 | 0.918 |
|  | CD3E | -0.036 | 0.411 | -0.009 | 0.837 | -0.045 | 0.313 | 0.043 | 0.348 |
|  | CD2 | -0.075 | 0.087 | -0.056 | 0.214 | -0.062 | 0.167 | 0.021 | 0.649 |
| Naive T-Cell | CCR7 | 0.015 | 0.741 | 0.065 | 0.148 | 0.007 | 0.872 | 0.104 | 0.023 |
|  | LEF1 | 0.014 | 0.755 | 0.038 | 0.406 | 0.042 | 0.343 | 0.031 | 0.504 |
|  | TCF7 | 0.057 | 0.198 | 0.086 | 0.055 | 0.170 | ** | 0.222 | *** |
|  | SELL | -0.131 | * | -0.116 | * | -0.041 | 0.358 | 0.050 | 0.275 |
| Effector T-Cell | CX3CR1 | -0.122 | * | -0.103 | 0.022 | -0.090 | 0.045 | -0.016 | 0.728 |
|  | FGFBP2 | -0.015 | 0.726 | -0.003 | 0.940 | -0.005 | 0.917 | -0.021 | 0.652 |
|  | FCGR3A | -0.253 | *** | -0.277 | *** | -0.162 | ** | -0.094 | 0.040 |
| Effector memory T-Cell | PDCD1 | 0.019 | 0.661 | 0.039 | 0.384 | 0.096 | 0.032 | 0.185 | *** |
|  | DUSP4 | 0.131 | * | 0.133 | * | 0.078 | 0.081 | 0.128 | * |
|  | GZMK | -0.087 | 0.049 | -0.061 | 0.171 | -0.072 | 0.110 | 0.008 | 0.865 |
|  | GZMA | -0.135 | * | -0.133 | * | -0.090 | 0.042 | -0.031 | 0.502 |
|  | IFNG | -0.045 | 0.312 | -0.042 | 0.348 | 0.061 | 0.173 | 0.101 | 0.027 |
| Resident memory T-Cell | CD69 | -0.125 | * | -0.115 | 0.010 | -0.071 | 0.112 | 0.013 | 0.782 |
|  | ITGAE | 0.040 | 0.360 | 0.036 | 0.430 | 0.140 | * | 0.116 | 0.011 |
|  | CXCR6 | -0.097 | 0.027 | -0.089 | 0.047 | -0.081 | 0.071 | -0.006 | 0.899 |
|  | MYADM | -0.109 | 0.013 | -0.110 | 0.015 | -0.006 | 0.892 | 0.051 | 0.269 |
| B cell | CD19 | 0.109 | 0.014 | 0.162 | ** | 0.020 | 0.652 | 0.111 | 0.015 |
|  | CD79A | 0.021 | 0.629 | 0.055 | 0.221 | -0.080 | 0.073 | -0.010 | 0.830 |
| Monocyte | CD86 | -0.275 | *** | -0.293 | *** | -0.156 | ** | -0.077 | 0.093 |
|  | CD115 (CSF1R) | -0.178 | *** | -0.172 | ** | -0.107 | 0.017 | -0.014 | 0.762 |
| TAM | CCL2 | -0.218 | *** | -0.220 | *** | -0.137 | * | -0.083 | 0.071 |
|  | CD68 | -0.201 | *** | -0.213 | *** | -0.154 | ** | -0.081 | 0.077 |
|  | IL10 | -0.163 | ** | -0.165 | ** | -0.097 | 0.029 | -0.028 | 0.539 |
| M1 Macrophage | INOS  (NOS2) | 0.009 | 0.847 | 0.011 | 0.809 | 0.067 | 0.134 | 0.089 | 0.051 |
|  | IRF5 | 0.012 | 0.795 | 0.017 | 0.709 | 0.101 | 0.024 | 0.125 | * |
|  | COX2 (PTGS2) | -0.003 | 0.948 | -0.013 | 0.773 | -0.057 | 0.200 | -0.041 | 0.373 |
| M2 Macrophage | CD163 | -0.164 | *** | -0.173 | ** | -0.119 | * | -0.041 | 0.375 |
|  | VSIG4 | -0.230 | *** | -0.243 | *** | -0.206 | *** | -0.141 | * |
|  | MS4A4A | -0.251 | *** | -0.269 | *** | -0.206 | *** | -0.136 | * |
| Neutrophils | CD66b (CEACAM8) | -0.010 | 0.827 | 0.000 | 0.997 | -0.019 | 0.673 | 0.005 | 0.916 |
|  | CD11b (ITGAM) | -0.122 | * | -0.119 | * | -0.018 | 0.689 | 0.075 | 0.100 |
|  | CCR7 | 0.015 | 0.741 | 0.007 | 0.873 | 0.065 | 0.148 | 0.104 | 0.023 |
| Natural killer cell | KIR2DL1 | 0.039 | 0.382 | 0.011 | 0.798 | 0.049 | 0.281 | 0.041 | 0.373 |
|  | KIR2DL3 | -0.058 | 0.189 | 0.062 | 0.163 | -0.044 | 0.332 | 0.098 | 0.033 |
|  | KIR2DL4 | -0.071 | 0.106 | -0.010 | 0.828 | -0.072 | 0.108 | 0.036 | 0.431 |
|  | KIR3DL1 | 0.075 | 0.088 | -0.036 | 0.425 | 0.096 | 0.033 | 0.011 | 0.819 |
|  | KIR3DL2 | -0.017 | 0.707 | 0.013 | 0.776 | -0.001 | 0.988 | 0.053 | 0.250 |
|  | KIR3DL3 | 0.063 | 0.151 | 0.015 | 0.730 | 0.078 | 0.082 | 0.034 | 0.458 |
|  | KIR2DS4 | 0.014 | 0.755 | 0.027 | 0.542 | 0.028 | 0.532 | 0.058 | 0.208 |
| Dendritic cell | HLA-DPB1 | -0.143 | * | -0.118 | * | -0.135 | * | -0.037 | 0.416 |
|  | HLA-DQB1 | -0.078 | 0.079 | -0.091 | 0.041 | -0.066 | 0.141 | -0.028 | 0.537 |
|  | HLA-DRA | -0.237 | *** | -0.177 | *** | -0.240 | *** | -0.109 | 0.017 |
|  | HLA-DPA1 | -0.192 | *** | -0.116 | * | -0.187 | *** | -0.042 | 0.355 |
|  | BDCA-1 (CD1C) | -0.115 | * | -0.158 | ** | -0.094 | 0.037 | -0.072 | 0.117 |
|  | BDCA-4 (NRP1) | -0.031 | 0.488 | -0.066 | 0.140 | -0.022 | 0.619 | 0.010 | 0.827 |
|  | CD11c (ITGAX) | 0.122 | * | 0.112 | 0.012 | 0.153 | ** | 0.247 | *** |
| Th1 | TBX21  (T-bet) | 0.048 | 0.276 | 0.124 | * | 0.072 | 0.110 | 0.218 | *** |
|  | STAT4 | -0.004 | 0.919 | 0.040 | 0.377 | 0.012 | 0.788 | 0.125 | * |
|  | STAT1 | -0.168 | ** | 0.039 | 0.384 | -0.177 | *** | 0.089 | 0.053 |
|  | IFNG (IFN-g) | -0.045 | 0.312 | 0.061 | 0.173 | -0.042 | 0.348 | 0.101 | 0.027 |
|  | TNF-a (TNF) | -0.018 | 0.682 | -0.057 | 0.199 | 0.012 | 0.789 | 0.007 | 0.877 |
| Th2 | GATA3 | -0.076 | 0.083 | 0.075 | 0.095 | -0.061 | 0.178 | 0.130 | * |
|  | STAT6 | 0.283 | *** | 0.233 | *** | 0.284 | *** | 0.251 | 0.000 |
|  | STAT5A | -0.024 | 0.586 | 0.101 | 0.023 | -0.002 | 0.961 | 0.198 | *** |
|  | IL13 | 0.044 | 0.314 | 0.037 | 0.413 | 0.057 | 0.210 | 0.084 | 0.068 |
| Tfh | BCL6 | 0.292 | *** | 0.235 | *** | 0.304 | *** | 0.237 | *** |
|  | IL21 | -0.041 | 0.353 | -0.007 | 0.870 | -0.038 | 0.394 | 0.043 | 0.351 |
| Th17 | STAT3 | 0.114 | * | 0.097 | 0.030 | 0.116 | 0.010 | 0.132 | * |
|  | IL17A | 0.019 | 0.665 | -0.033 | 0.468 | 0.037 | 0.414 | -0.022 | 0.632 |
| Treg | FOXP3 | -0.087 | 0.048 | -0.021 | 0.645 | -0.068 | 0.129 | 0.073 | 0.113 |
|  | CCR8 | -0.156 | ** | -0.113 | 0.012 | -0.136 | * | -0.031 | 0.500 |
|  | STAT5B | 0.202 | *** | 0.323 | *** | 0.216 | *** | 0.352 | *** |
|  | TGFB1 (TGFb) | -0.090 | 0.042 | -0.067 | 0.136 | -0.086 | 0.057 | -0.026 | 0.573 |
| T cell exhaustion | PDCD1 (PD-1) | 0.019 | 0.661 | 0.096 | 0.032 | 0.039 | 0.384 | 0.185 | *** |
|  | CTLA4 | 0.027 | 0.541 | 0.049 | 0.274 | 0.061 | 0.180 | 0.151 | ** |
|  | LAG3 | 0.009 | 0.841 | 0.075 | 0.095 | 0.022 | 0.633 | 0.139 | * |
|  | HAVCR2  (TIM-3) | -0.270 | *** | -0.172 | ** | -0.292 | *** | -0.101 | 0.027 |
|  | GZMB | -0.107 | 0.016 | -0.033 | 0.459 | -0.108 | 0.016 | 0.041 | 0.373 |

TAM, tumor-associated macrophage; Th, T helper cell; Tfh, Follicular helper T cell; Treg, regulatory T cell; Cor, R value of Spearman’s correlation; None, correlation without adjustment. Purity, correlation adjusted by purity.

* *p* < 0.01; ** *p* < 0.001; *** *p* < 0.0001.

**Figure S1**

**
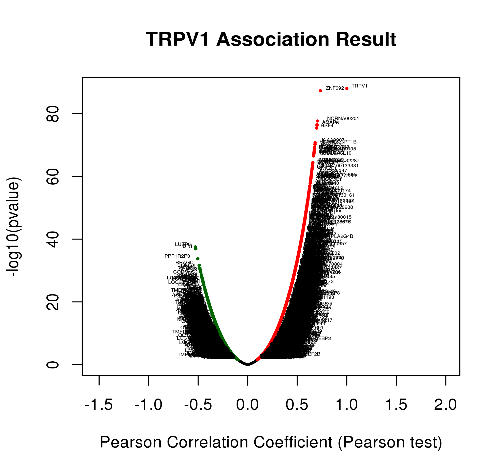

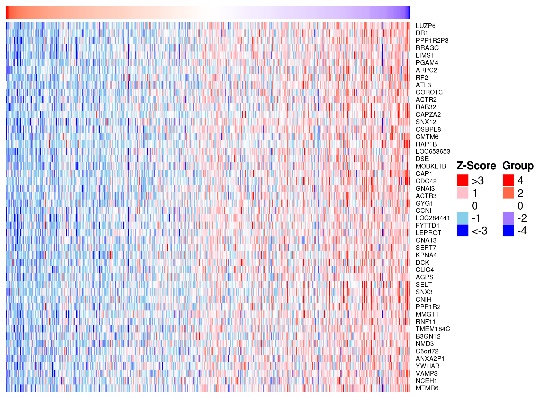

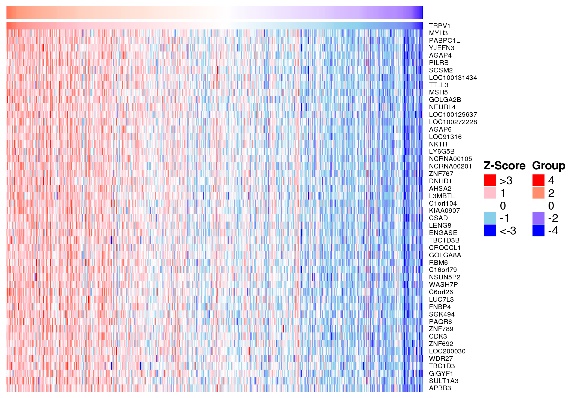

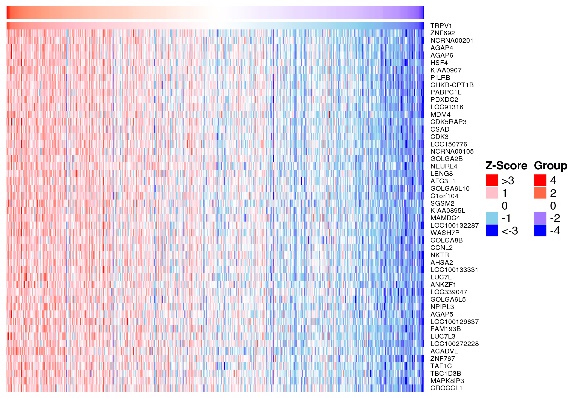

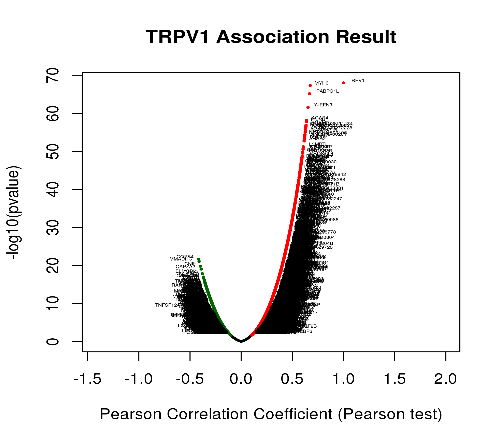

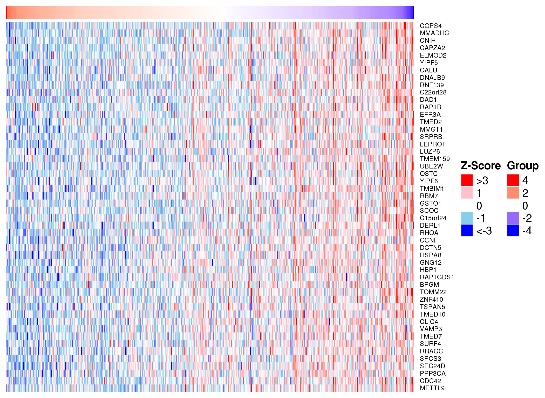
**

**Negatively correlated genes**

**Positively correlated genes**

LUSC

LUAD

**Figure S2**


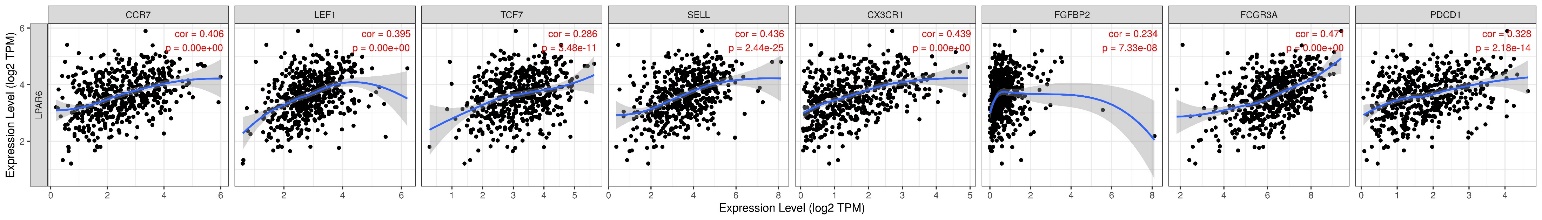

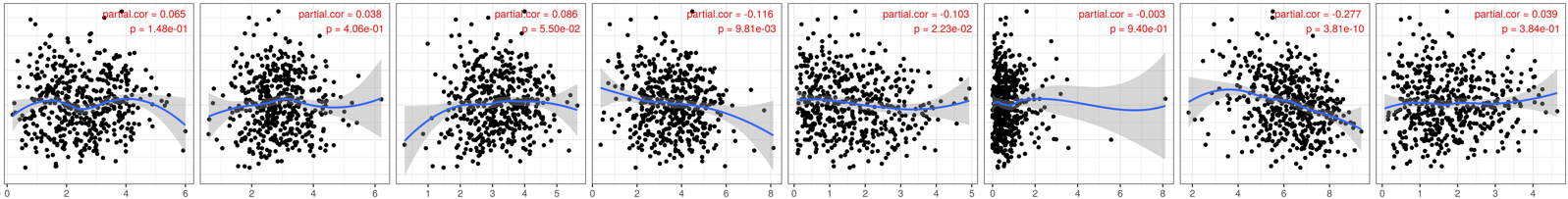


**A B C D E F G H**

**
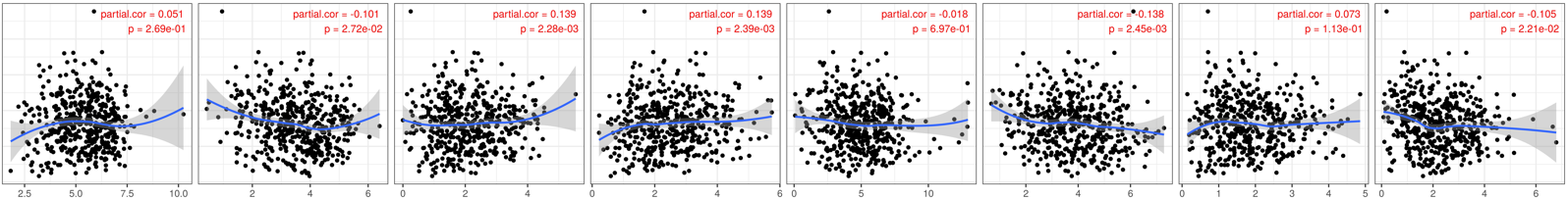

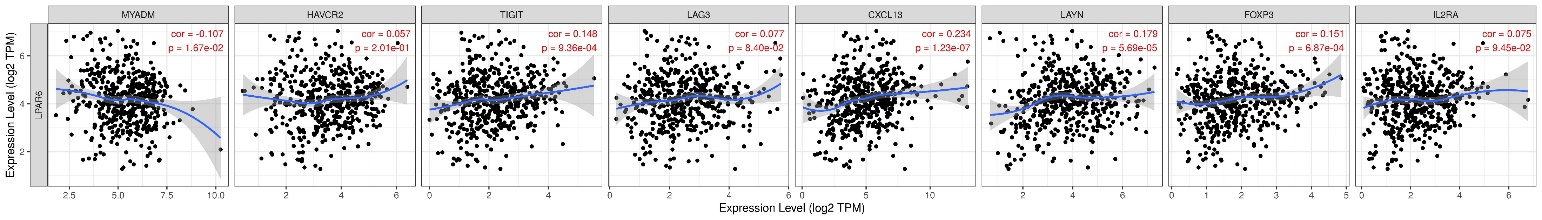
**


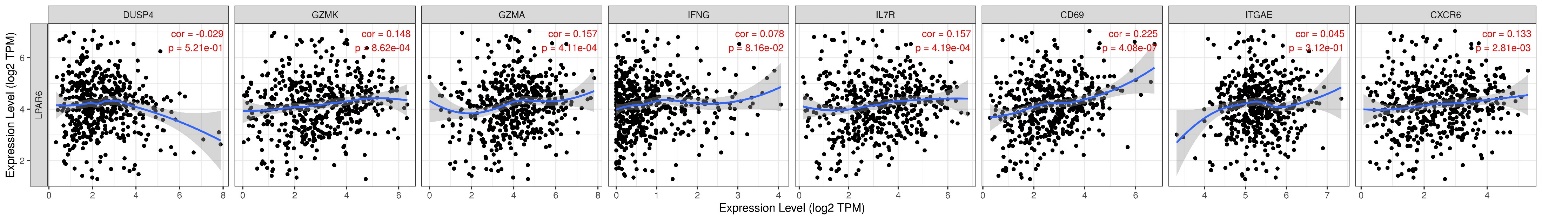

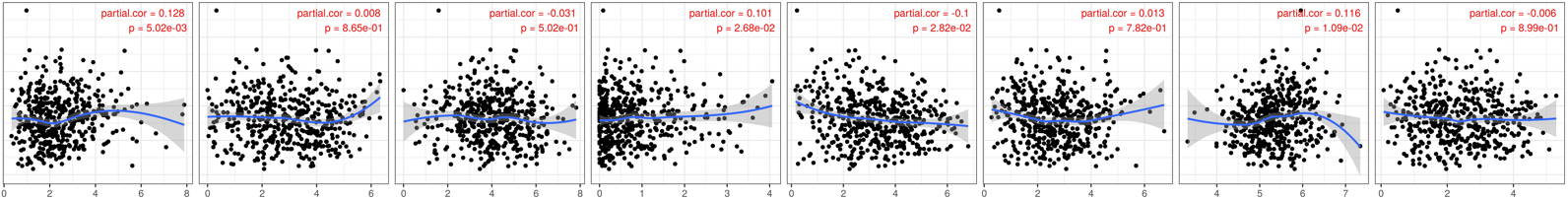


**AN AO AP AQ AR AS AT AU**


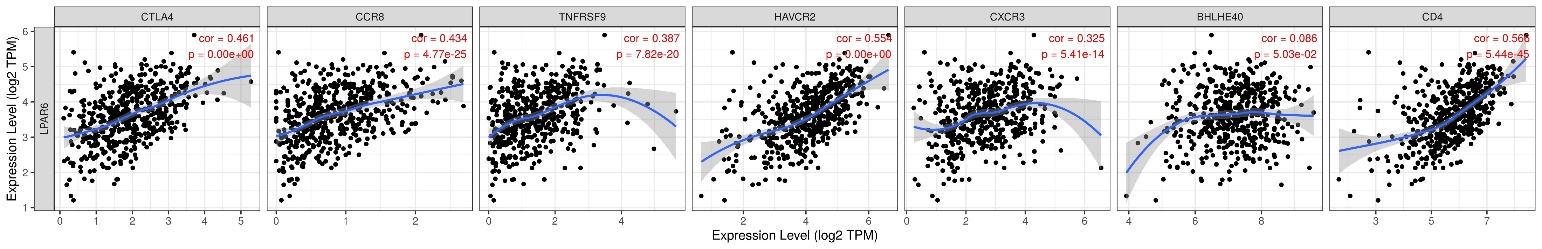

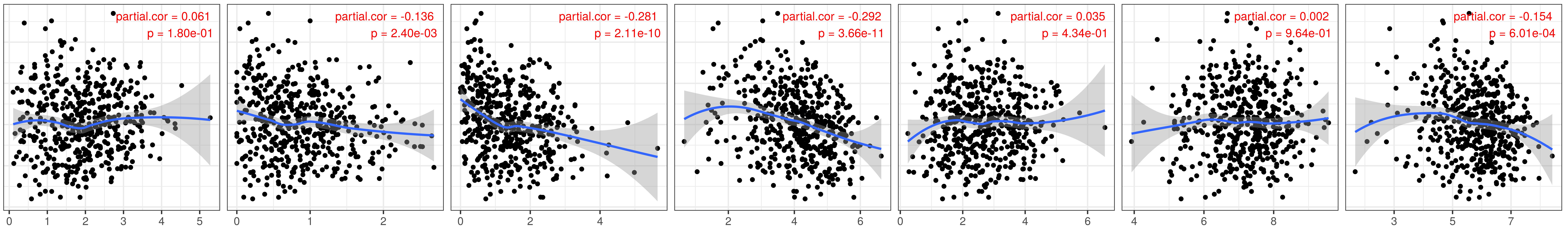


**Y Z AA AB AC AD AE**


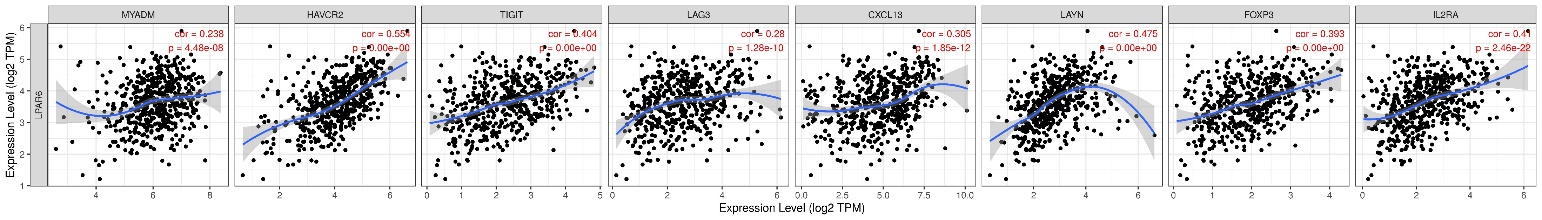

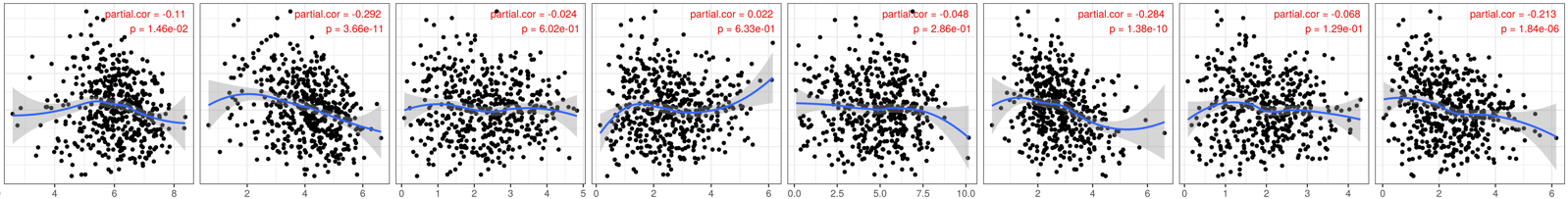


**Q R S T U V W X**


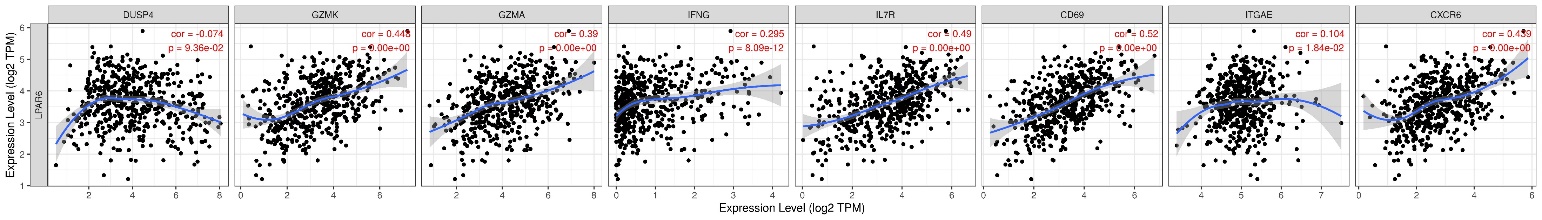

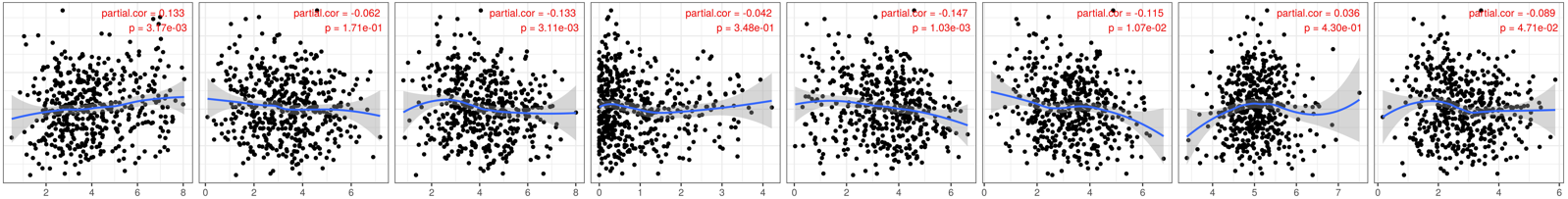


**I J K L M N O P**


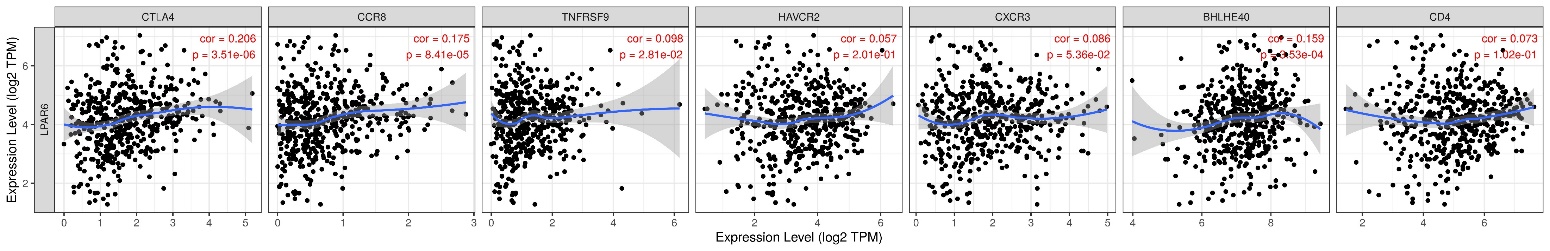

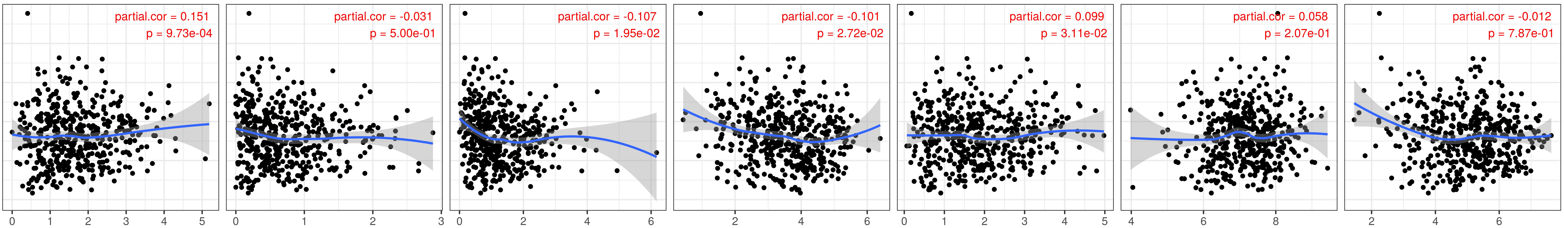


**BD BE BF BG BH BI BJ**

LUSC

**AV AW AX AY AZ BA BB BC**


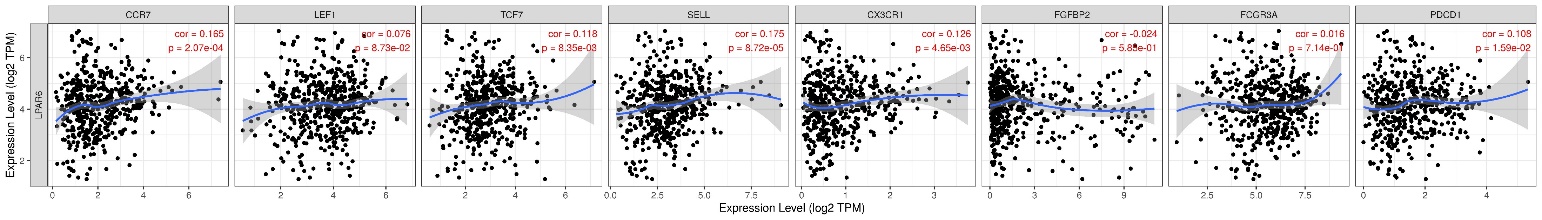

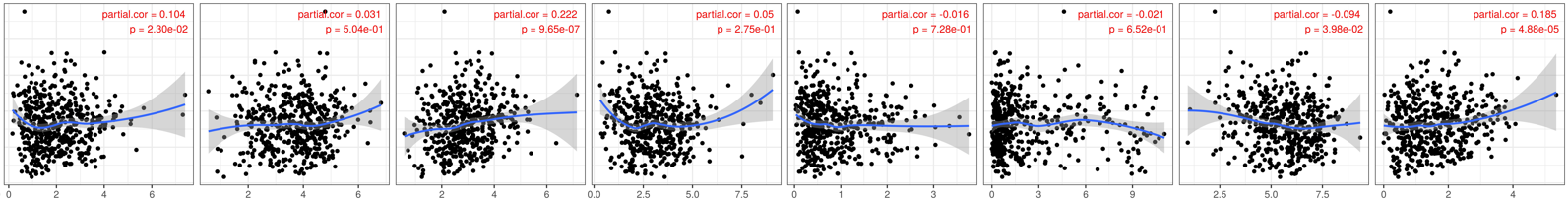


**AF AG AH AI AJ AK AL AM**

LUAD

**Figure S3**

**A B C D**

**Type 2 T helper cell**

**Normal**

**Tumor**

**Normal**

**Tumor**

**Regulatory T cell**

**M N O P**

**I J K L**

**Activated dendritic cell**

**MDSC**

**E F G H**

LUSC

**Eosinophil**

**Normal**

**Tumor**

**CD56bright natural killer cell**

**Effector memory CD4 T cell**

**Normal**

**Tumor**

**Tumor**

**Normal**

**Tumor**

**Normal**

**Activated B cell**

**Normal**

**Tumor**

**Normal**

**Tumor**

**Figure Legends**

**Figure S1 | Differentially expressed genes in correlation with TRPV1 and heat maps of positively and negatively correlated genes with TRPV1 in LUAD and LUSC were analyzed by Pearson test.** Red indicates positive and blue indicates negative.

**Figure S2 | Correlation analysis between TRPV1 expression and various T cell marker sets in LUAD (A-AE) and LUSC (AF-BJ) patient cohort.** Scatterplots of correlations between TRPV1 expression and gene markers of Naive T-Cell (CCR7, LEF1, TCF7, SELL) (A-D), Effector T-Cell (CX3CR1, FGFBP2, FCGR3A) (E-G), Effector memory T-Cell (PDCD1, DUSP4, GZMK, GZMA, IFNG) (H-L), Central memory T-Cell (CCR7, SELL, IL7R) (A, D, M), Resident memory T-Cell (CD69, ITGAE, CXCR6, MYADM) (N-Q), T cell exhaustion (HAVCR2, TIGIT, LAG3, PDCD1, CXCL13, LAYN) (R-T, H, U-V), Resting Treg (FOXP3, IL2RA) (W, X), Effector Treg (FOXP3, CTLA4, CCR8, TNFRSF9) (W, Y-AA), Th1-like (HAVCR2, IFNG, CXCR3, BHLHE40, CD4) (AB, L, AC-AE) in LUAD; (AF–BJ) Scatterplots of correlations between LPAR6 expression and gene markers of Naive T-Cell (CCR7, LEF1, TCF7, SELL) (AF-AI), Effector T-Cell (CX3CR1, FGFBP2, FCGR3A) (AJ-AL), Effector memory T-Cell (PDCD1, DUSP4, GZMK, GZMA, IFNG) (AM-AQ), Central memory T-Cell (CCR7, SELL, IL7R) (AF, AI, AR), Resident memory T-Cell (CD69, ITGAE, CXCR6, MYADM) (AS-AV), T cell exhaustion (HAVCR2, TIGIT, LAG3, PDCD1, CXCL13, LAYN) (AW-AY, AM, AZ-BA), Resting Treg (FOXP3, IL2RA) (BB-BC), Effector Treg (FOXP3, CTLA4, CCR8, TNFRSF9) (BB, BD, BE-BF), Th1-like (HAVCR2, IFNG, CXCR3, BHLHE40, CD4) (BG, AQ, BI-BJ) in LUSC.

**Figure S3 | Correlation analysis between TRPV1 expression and various immune cells in normal and tumor tissue of LUSC.** Scatterplots of correlations between TRPV1 expression and Regulatory T cell (A, B), Type 2 T helper cell (C, D), MDSC (E, F), Activated dendritic cell (G, H), Activated B cell (I, J), Eosinophil (K, L), Effector memory CD4 T cell (M, N), CD56bright natural killer cell (O, P) in the normal and tissue of LUSC.

**Abbreviation**

| LUAD | Lung adenocarcinoma |
| --- | --- |
| TRPV1 | Transient receptor potential cation channel, subfamily V, member 1 |
| TILs | Tumor-infiltrating lymphocytes |
| TCGA | The Cancer Genome Atlas |
| LUSC | Lung squamous cell carcinoma |
| HR | Hazard ratio |
| OS | Overall Survival |
| PFS | Progression-free survival |
| TIICs | Tumor infiltrating immune cells |
| DEG | Differential expressed genes |
| OS | Overall survival |
| TME | Tumor microenvironment |
|  |  |
